# Calcineurin-nuclear factor for activated T cells (NFAT) signaling in pathophysiology of wound healing

**DOI:** 10.1186/s41232-021-00176-5

**Published:** 2021-08-18

**Authors:** Takahiro Manabe, Heamin Park, Takashi Minami

**Affiliations:** Division of Molecular and Vascular Biology, IRDA, Kumamoto University, 2-2-1 Honjyo Chuo-ku, Kumamoto, 860-0811 Japan

**Keywords:** Wound healing, NFAT, Coagulation, Angiogenesis, Inflammation

## Abstract

Wound healing occurred with serial coordinated processes via coagulation-fibrinolysis, inflammation following to immune-activation, angiogenesis, granulation, and the final re-epithelization. Since the dermis forms critical physical and biological barriers, the repair system should be rapidly and accurately functioned to keep homeostasis in our body. The wound healing is impaired or dysregulated via an inappropriate microenvironment, which is easy to lead to several diseases, including fibrosis in multiple organs and psoriasis. Such a disease led to the dysregulation of several types of cells: immune cells, fibroblasts, mural cells, and endothelial cells. Moreover, recent progress in medical studies uncovers the significant concept. The calcium signaling, typically the following calcineurin-NFAT signaling, essentially regulates not only immune cell activations, but also various healing steps via coagulation, inflammation, and angiogenesis. In this review, we summarize the role of the NFAT activation pathway in wound healing and discuss its overall impact on future therapeutic ways.

## Background

Wound healing occurred with a coordinative process of three phases: inflammation, proliferation, and remodeling. When tissues are injured, they initially occur clot formation to stop the bleeding following inflammation. In this phase, hemostatic balance and immune control are critical for precise wound healing. Macrophage and neutrophils migrate to the wound site to perform infection control, and platelets release several growth factors involving vascular endothelial growth factor (VEGF), fibroblast growth factor (FGF), and platelet-derived growth factor (PDGF) [1]. These factors can lead to the next proliferation phase causing re-epithelialization, granulation, and angiogenesis. Re-epithelialization starts within a few hours after injury [2]. Clots and damaged stroma are removed from the skin, mediated by the proliferation and migrations of epithelial cells and keratinocytes. In these cells, tonofilament and actin filament are reorganized. These disrupt hemidesmosome links between the epidermis and the basement membrane, which allows dynamic movement of epidermal cells. Epithelial cells also release growth factors, including epithelial growth factor (EGF), which lead to re-epithelization and granulations [3]. The final phase, remodeling, occurred via the combinations among at least mesenchymal cells, vessel-forming endothelial cells and pericytes, and fibroblasts. These cells activate in the presence of several cytokines that reveal higher migration capacities and extracellular matrix production in granulation tissue [4].

Angiogenesis plays a key role in wound healing. It is needed to transport oxygen and nutrition, vital for cells, to the wound site. By migration and proliferation of these cells, a wound in tissue is filled. After the proper angiogenesis process, granulation tissue is replaced by an acellular scar due to cell apoptosis. But at last, no longer needed abundant angiogenic blood vessels are regressed [5]. These angiogenesis and tissue-granulation processes overlap in time, and the coordination of each step is essential for wound healing.

Interestingly, calcium homeostasis is related to wound healing status. For example, calcitonin treatment upregulates calcium levels in wound sites. The upregulated calcium leads to enhanced wound healing in rabbits [6] and rats [7]. Low-calcium diets resulted in delayed wound healing in vitamin D receptor knockout mice [8]. Moreover, calcium influx stimulates through phospholipase C pathways that lead several downstream signal axis, including protein kinase C and calmodulin. Calcineurin activation also occurred following NFAT nuclear localization in many cell types. NFAT is a famous immune- or inflammatory regulated transcription factor that obtains a family with several types of isoforms: NFAT1 to 4. Calcineurin-NFAT inhibitor, cyclosporine A (CsA), is the most famous immunosuppressive drug after organ transplantations. Moreover, it is known that downregulation of calcineurin attenuates neural inflammation via tau hyperphosphorylation in neuronal cells [9]. In this review, we are going to focus on the NFAT signaling in multiple wound healing steps as shown below related to several pathologies.

## Main text

### NFAT signaling in coagulations

**Fig. 1:**
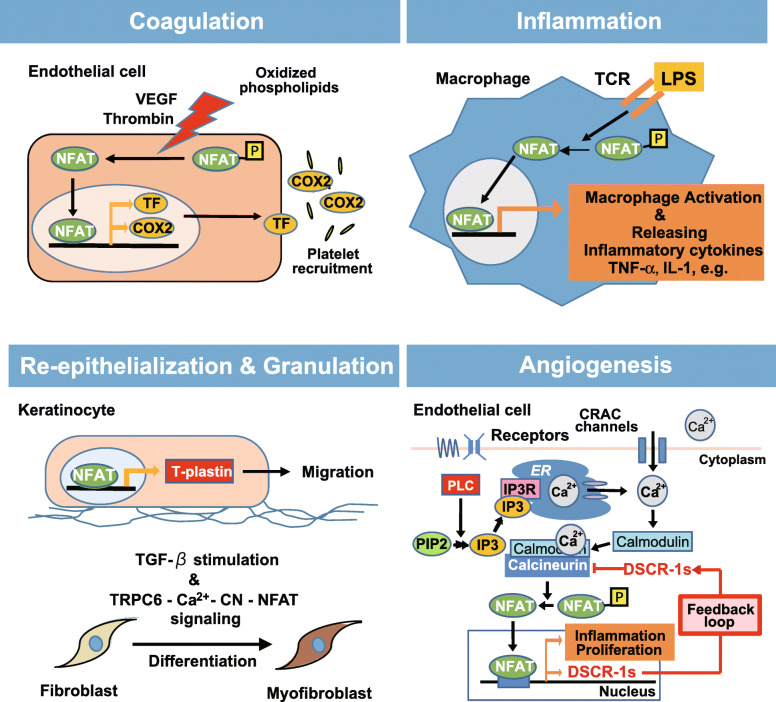
Schematic illustration of NFAT-mediated wound healing processes. Wound healing steps are summarized following the orders of coagulation, inflammation, and the subsequent re-epithelization, granulation, and angiogenesis. CRAC; calcium release-activated calcium, PLC; phospholipase C, PIP2; phosphatidylinositol 4,5-bisphosphate, IP3; inositol trisphosphate, ER; endoplasmic reticulum

The coagulation process consists of platelet adhesion, secretion, and aggregation. Wound-initiated coagulation, extrinsic pathway, is started by tissue factor (TF) expression on the perivascular tissue surfaces. TF is a transmembrane glycoprotein that binds to factor VII following the proteolytic activation (FVIIa). Such TF-FVIIa complex activates the downstream coagulation pathways and finally leads to thrombin burst [10]. Since TF promoter has a functional NFAT-binding site, TF expression is induced by VEGF [11] and oxidized phospholipids [12] via calcineurin-NFAT signaling. However, NFAT overactivation may destabilize the coagulation balance. NFAT can also induce cyclooxygenase (COX) 2. It has been reported that cox2 and its inhibition cause complex outcomes, blocking or enhancing clot formation (Fig. 1). Moreover, clinical use of cyclosporine (CsA) was suggested to be associated with an increased risk of thromboembolic complications. Platelets treated with CsA in vitro induced procoagulant activity. However, compared to CsA, tacrolimus (also known as FK506) significantly decreased the propensity to form thrombus of cardiac allograft vasculopathy. Nevertheless, these immunosuppressive drugs are not entirely dependent on NFAT inhibition. Thus, the molecular mechanisms underlying these complex effects remain unresolved.

### NFAT signaling in inflammations

Inflammation occurs as a necessary process for wound healing. In this phase, innate immune cells; macrophages and mast cells, predominantly activate to control infection and enhance many cell proliferation. Macrophages infiltrate to the wounded area for phagocytosing specific proteins of extracellular matrix from foreign particles or bacteria via binding to macrophage’s integrin receptor. In such innate immunity, NFAT signaling is known to have critical roles. For example, NFAT-bound enhancers on the IL-3 gene locus promote macrophage differentiation from stem cells [13], which proceed the inflammation via releasing TNF-α. Macrophages are known to secrete interleukin (IL)-2 or 6, which introduces cell proliferation by themselves. Especially macrophages in the healing process, phenotype switching from classically inflamed (M1) to “wound healing macrophage (M2-like)” occurred [1]. In the early wounds, stimulation of Toll-like receptors (TLR) 1, 2, and 4 by lipopolysaccharide (LPS) induces macrophage activation under the nuclear localization of NFAT3 and 4 (Fig. 1). As the wound healing progresses, macrophages transform into wound healing macrophages by a complex of factors, IL-10, glucocorticoids, prostaglandins, metabolites, and the process of efferocytosis [14].

It has been reported that mast cells are required for proper wound healing. Mast cells accumulate within 24 h from the injury and release cytokines, VEGF and histamine, which are necessary to proliferate for angiogenic endothelial cells and mast cells by themselves, respectively [15]. In activated mast cells, the *Hif*-1α gene is upregulated via NFAT binding to the promoter region, which can induce the downstream VEGF expression via phosphatidylinositol 3-kinase-dependent manner.

### NFAT signaling in re-epithelialization and granulations

To reduce the infection risks of the wound area, the so-called re-epithelialization, instant barrier is formed within a few hours by migration and proliferation [16]. In keratinocytes, NFAT activation is reported to accelerate T-typed plastin (T-plastin) synthesis and promote migration (Fig. 1). T-PLASMIN is an ACTIN binding protein belonging to the fimbrin family and helps the assembly and stabilization of the actin fiber network [17]. In contrast, NFAT inhibitor treatment decreases the migration of keratinocytes [18]. It suggests the direct correlation between CsA treatment and the dysregulation of wound healing in patients [19].

Although re-epithelialization is completed, granulation is still ongoing under the epidermis. Granulation tissue appears within 3–5 days after the injury [20]. In this phase, due to the maximal activation of mesenchymal stromal cells and fibroblasts, the migration capacities and following the ECM production are increased [5]. Fibroblasts from calcineurin B knockout mice were reported to indicate “activated phenotypes” with high migration capacity, increased collagen secretion, and remodeling [21]. Moreover, these fibroblasts decreased the response to TGF-β. Surprisingly, CsA treatment alone failed to change the fibroblast-mediated collagen secretion [22]. The precise mechanism of the differences between CsA and the calcineurin null mutated mice is not well understood, but at least it can be caused via the non-genomic effects under the CsA treatment or the different CsA inhibitory thresholds between the downstream calcineurin-mediated transcription factors, NFAT, and myocyte enhancer factor (MEF)2.

During the granulation process, tissue urgently regenerates in the lack of blood circulation. Thus, hypoxic conditions usually happen, and it is believed that the relieving hypoxia-induced factor (HIF) 1/2α induction and the stabilization are critical for the proper wound healings [23]. Shortage of HIF-1α can be caused to form an intractable ulcer, whereas HIF-1α overexpression in dermal fibroblasts, in turn, can be led to fibrotic disease such as keloid formation [24]. Moreover, it is reported that hypoxia-mediated activation of NFAT1 occurs through HIF-2α, but it does not occur through HIF-1α. Also, knockdown or chemical inhibition of HIF-2α inhibited hypoxia-induced NFAT1 nuclear translocation. Moreover, overexpression of HIF-2α, not HIF-1α, increased NFAT1 transcriptional activity in pulmonary fibroblasts. There are two possibilities of activating NFAT1 signaling by HIF-2α. One of the possibilities is that HIF-2α induces the expression of positive components in NFAT signaling or the genes that upregulate NFAT signaling. It has been reported that hypoxia increases NFAT1 expression in HIFs [24], and the activation of HIF-2α increases mRNA expression levels of NFAT1 and 4 [25]. Another possibility of activating NFAT signaling by HIF-2α is direct interaction of HIF-2α with NFAT1. Further elucidations such as protein-protein interaction analysis or reporter assays would be needed to uncover the detailed mechanism of the correlation between hypoxia and NFAT signaling.

Myofibroblast formation is necessary for proper wound healing. TGF-β stimulates the differentiation from the original fibroblasts, which caused contraction of wound sites [26]. Myofibroblast has bold microfilament bundles including actin, and it shows not only higher secretion of ECMs, but also contractile features like smooth muscle, which contributes to wound shrinkage [27]. The calcium channel, TRPC6, mediated-NFAT signaling can involve the myofibroblast differentiation process [28]. Deleted TRPC6 delayed wound healing, whereas such the phenotype was rescued by the administration of constitutively active calcineurin. Following these reports, CsA administration leads to a significant decrease in both wound contraction levels and accumulation of hydroxyproline, the marker for reparative collagen deposition [29], suggesting that NFAT signaling not only promoted the inflammation of wounds, but also proceeded to the re-epithelialization and granulation step during the proper healing process. Interestingly, it has been reported that microamperage electrical stimulation (MES) increases NFAT activation, resulting in increased wound healing capacities [30, 31]. MES can increase the granulation and accelerated the re-epithelialization process via the increase of dermal collagen production. Electrical stimulation also increased the proliferation of myofibroblasts via the NFAT activation [32]. Collectively, these findings suggest that MES and the resulting NFAT activation can be a new therapeutic way against pathological dermal wounds.

### NFAT signaling in angiogenesis

Cell proliferation under the tissue regeneration process needs enough oxygen and nutrient supplies, so that neo-vessel formation from existing vessels, angiogenesis, is necessary for wound healing. Angiogenesis is stimulated by many angiocrine factors which are predominantly secreted from inflammatory cells. Thus, inflammation and the following angiogenesis are tightly connected. Exquisite coupling of inflammation and angiogenesis would be critical for adequate healings. In this theory, patients treated with the anti-angiogenesis drug, such as bevacizumab, or diabetes patients appear to have delayed wound healing, mainly due to dysregulation of angiogenesis [33, 34].

We have previously reported both VEGF and thrombin induce NFAT nuclear localization. The following upregulation of various NFAT downstream angiogenesis-related genes involves TF, Early growth response (Egr)2/3, Cox2, bone morphogenic protein (Bmp)2/4, and Cxcr4/7 [35, 36]. Moreover, VEGF but not TNF-α signals preferentially activate NFAT rather than NF-κB. Then, they induce various inflammatory-related genes: vascular and intercellular cell adhesion molecule (Vcam and Icam) 1, E-selectin, and monocyte chemotactic protein (Mcp) 1. Not only in in vitro cultured cells, NFAT activations are also indispensable for angiogenesis-related proper development in mice. NFAT2 null mutation indicates embryonic lethality in embryonic days (E)13.5–17.5 due to failure of the artery and coronally endocardium-mediated valve formation [37]. Combined null mutation of NFAT3 and 4 also revealed lethality at E11.5 by the disorganization of growing vessels leading to failure of fully assemble matured vessels [38]. Calcium-calcineurin-responsive NFAT is recognized to NFAT1 to 4, and all subtypes are expressed in at least primary cultured endothelium from various organs. Thus, it is still needed to verify why such distinct phenotypic differences are observed among each NFAT null mutation in mice. To uncover the mechanisms against the phenotypic differences, several advanced comprehensive approaches, genome-wide chromatin immunoprecipitation (ChIP)-seq by using each specific NFAT antibody to verify the specified downstream targeted genes, and the targeted proteomics to identify the physical protein-protein interaction with each NFAT [39] are available with recent technical proceedings.

**Fig. 2:**
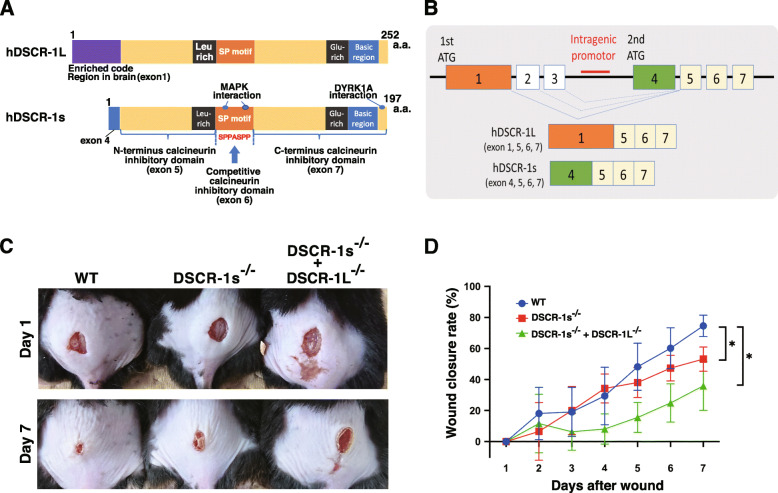
DSCR-1-mediated wound healing. **A** Human DSCR-1L and DSCR-1s protein. a.a. amino acids. **B** Splicing patterns of human *DSCR-1* genome. The schematic data is modified from ref. [35]. **C** The representative wound healing patterns after days 1 and 7. **D** Quantified wound closure rate. Data was shown as the mean and standard deviations, *N* = 3. *P < 0.05 compared with WT. WT wild-type control, Dscr-1s^−/−^ mouse Dscr-1 short isoform-specific null mutation, Dscr-1L^−/−^ mouse Dscr-1 long isoform-specific null mutation

NFAT hyperactivation or sustained activation usually occurs for destabilization of cell stability. As an adjusted feedback molecule for NFAT activation in endothelial cells, we have first identified and reported the specific gene, the Down syndrome critical region (Dscr)-1 [35]. Dscr-1 stable expression in the endothelium autoinhibits the NFAT nuclear localization, resulting in attenuated tumor growth, metastasis to the lung, and septic inflammation [40, 41] (Fig. 1). Dscr-1 predominantly encodes two types of isoform. Long isoform (Dscr-1L) contains exon1, 5 to 7, and short isoform (DSCR-1s) contains exons 4 to 7 (Fig. 2A and B). However, typically in wound healing processes, the functional differences between long and short Dscr-1 isoforms have not yet been studied. To that end, we have succeeded to generate each isoform-specific knockout mouse via TALEN and CRISPR/Cas9 technology. As a primal test, we showed a part of data that short isoform null mutation, Dscr-1s^−/−^, delayed wound healing ~ 30% compared to the wild-type control. In contrast, Dscr-1s and L combined null mutation caused the wound healing delay ~ 60% than control (Fig. 2C and D). It has been reported that loss of Dscr-1 caused hyperactivation of NFAT. Based on this knowledge, Dscr-1 knockout mice may impair inflammation and the subsequent angiogenesis relay process via hyperactivation of NFAT. Further defined wound healing data with isoform-specific Dscr-1 knockout mice would be available and they will be useful for the discussion of current dermis ointment with CsA, tacrolimus, and sirolimus, an aspect of safety and effectiveness with angiogenesis.

## Conclusions

Spatial and temporal dysregulation or dysfunction of the wound healing process causes various symptoms involving fibrosis, keloid formation, and intractable ulcer that might lead to feeling down due to worsening changes of visual or un-visual aspects of complications in the body. Wound healing processes are involved in multiple steps as discussed above. We realized calcium signaling and the following calcineurin-NFAT activation axis are correlated in all of these healing processes (Fig. 1).

Classically, NFAT-mediated gene upregulations lead to well-known T cell differentiations and activation, but most recently, many reports also showed the NFAT activity correlates with innate immune cell activation, bone metabolism, angiogenesis, and tissue regenerations [42]. NF-κB, we felt, is the most famous or studied transcription factor for controlling inflammation-mediated cell growth or apoptosis. These critical functions need to feedback control system, such as I-κB, to maintain homeostasis [43]. Similarly, our mainly identified DSCR-1 indeed works as a feedback modulation for NFAT overactivation. Dscr-1 was initially named as the location of the 21th chromosome, and currently, it was uncovered that it really correlates with Down syndrome patients mediating the strong resistance of the solid tumor burden and the malignancy [44]. Moreover, DSCR-1 stable expression protects the mortality of septic severe inflammations and cytokine storms [40].

During the writing of this review, people in the world suffered from the pandemic of SARS-COV2, COVID-19. One of the reasons for the unexpected malignancy of COVID-19 is an immune uncontrolled cytokine storm, lead by the over-secretion of IL-6, TNF-α, and IFNγ, e.g., [45]. These cytokines are known to stimulate immunity and inflammation in our body. Moreover, VEGF secreted from lungs may correlate with the morbidity and mortality of SARS-COV2, as shown in detail in sepsis patients [46]. It has been reported that non-structural protein 1 of SARS-COV2 can be interacted with the immunophilin modulator domain of calcineurin, which induces the IL-2 expression in macrophages [47]. CsA treatment may work in not only the immunosuppression but also the virus replication inhibition [48]. Taken together, it will be a vital candidate for NFAT activation and the accurate control ways, for further drug screenings or future therapy against the SARS-COV2.

## Data Availability

The datasets during and/or analyzed during the current study are available from the corresponding author on reasonable request.

## Abbreviations

NFAT: Nuclear factor of activated T cell
VEGF: Vascular endothelial growth factor
FGF: Fibroblast growth factor
PDGF: Platelet-derived growth factor
EGF: Epithelial growth factor
CsA: Cyclosporine A
TF: Tissue factor
COX: Cyclooxygenase
IL: Interleukin
TLR: Toll-like receptors
LPS: Lipopolysaccharide
MEF: Myocyte enhancer factor
HIF: Hypoxia-induced factor
MES: Microamperage electrical stimulation
Egr: Early growth response
Bmp: Bone morphogenic protein
Cxcr: Chemokine receptor chemokine (C-X-C motif) receptor
TNF-α: Tumor necrosis factor-α
NF-κB: Nuclear factor kappa-light-chain-enhancer of activated B cells
Vcam: Vascular cell adhesion molecule
Icam: Intercellular cell adhesion molecule
Mcp: Monocyte chemotactic protein
ChIP: Chromatin immunoprecipitation
DSCR-1: Down syndrome critical region-1
SARS-COV2: Severe acute respiratory syndrome coronavirus 2
COVID-19: Coronavirus disease 2019
IFN: Interferon
